# Personalized Care in the UAE : A Study on Precision Medicine Awareness and Accessibility among the general population

**DOI:** 10.12688/f1000research.163687.1

**Published:** 2025-06-27

**Authors:** Jisha Myalil Lucca, Alhammadi Salama, Basant Abdalla, Jeswin Baby

**Affiliations:** 1 College of pharmacy, Gulf medical university, Ajman, United Arab Emirates; 2 Caritas Hospital & Institute of Health Sciences,, Kottayam, Kerala, India

**Keywords:** Precision Medicine, Pharmacogenomics, Genetic Testing, Public Awareness, UAE

## Abstract

**Background:**

Precision medicine is an emerging approach that tailors treatments based on an individual’s genetic profile. The UAE has made significant strides in this field through initiatives like the National Genomics Strategy and the Emirati Genome Program. However, public awareness and engagement remain key challenges.

**Purpose:**

This study assesses awareness, acceptance, and utilization of precision medicine among UAE residents.

**Methods:**

A cross-sectional online survey was conducted using a snowball sampling method, The survey collected demographic data, health status, knowledge, and experiences with precision medicine. Descriptive statistics and chi-square tests were used to analyze associations.

**Results:**

Most participants (94.3%) were under 50 years old, 62.5% were female, and 60.0% held a bachelor’s degree. Awareness of precision medicine was moderate (55.3%), with higher familiarity among females and students. While 40% believed its main benefit was optimizing drug effectiveness, 38.5% viewed it as crucial for preventing adverse drug reactions. Family and friends (29.5%) were the primary sources of information, yet 25.5% had never heard of precision medicine. Awareness of insurance coverage was low, with 59.0% uncertain about their policy. Genetic testing participation was associated with education level (p < 0.05). Acceptance of precision medicine was higher among individuals with chronic illnesses (p = 0.004). Familiarity scores varied significantly by occupation (p < 0.001) and income (p = 0.004), with higher-income individuals showing greater awareness. Males had a broader range of practice scores (p = 0.003), and individuals with chronic conditions were more aware of precision medicine (p = 0.023).

**Conclusion:**

Despite advancements, public engagement with precision medicine remains limited. Targeted educational initiatives, improved accessibility, and increased awareness of insurance coverage may enhance adoption and utilization.

## Introduction

Precision medicine, a novel approach in healthcare, provides an optimised targeted treatment to individual patients based on genetic composition, environmental factors, and lifestyle. ^1, 2^ It creates a revolutionary change in the treatment of rare and difficult to treat diseases by replacing the conventional one-size-fits-all treatment approach. ^3^ According to FDA “Personalised medicine, sometimes called individualised or precision medicine, is an evolving field in which physicians use diagnostic tests to determine which medical treatments will work best for each patient or use medical interventions to alter molecular mechanisms that impact health. By combining data from diagnostic tests with an individual’s medical history, circumstances, and values, health care providers can develop targeted treatment, and prevention plans with their patients.” ^4^ The main aim of precision medicine is to pair the treatment with unique characteristics of the patient and to enhance the safety and efficacy of medications. Beyond cancer treatment, precision medicine impacts extend in treating asthma, infectious diseases, connective tissue diseases, cardiovascular diseases, obesity and diabetes. ^2, 3, 5, 6^ Precision medicine have been shown to decrease the healthcare expenses and minimise physicians’ burden, foster more personalised patient care. ^7^ According to the FDA precision medicine report in 2022 a total of 12 new drugs identified as personalised medicines and 5 new gene or cell-based therapies were approved. ^4^

Health care system in the middle east is also pawing inroads into précised and genetic medicine. By implementing genetic medicine, the Middle East can prioritise the treatment of high prevalence chronic diseases, rare genetic and orphan disorders in the region. ^8^ Precision medicine market in the Middle East and Africa is set to expand at CAGR of 9.96 percent to a revenue worth of US$2.51 billion by 2023. ^9^ From 2023 to 2028, the precision medicine market in the MEA region is expected to expand at an average CAGR of 11.32%. Due to the market’s size, it is expected to increase from USD 4.98 billion in 2023 to USD 8.52 billion by 2028. ^9^

In March 2023, UAE launched a national genomics strategy to enhance the advancement of preventive medicine in the country. The Emirati Genome Program is a foundational project within the National Genome Strategy, which aims to use genomic data to enhance public health among UAE nationals. ^10, 11^ The first Personalized Precision Medicine Program for oncology was launched by the Abu Dhabi Department of Health. ^10, 11^ The main goal of the programme is to provide healthcare professionals with high-quality information that will enable them to offer advanced diagnostics, customized treatment options, and prevention programmes that are tailored to each individual’s unique genetic composition. ^10, 11^ It will also help in the development of innovative treatments for uncommon and chronic illnesses as well as improved prediction and prevention of current and future genetic diseases.

UAE, leading in adapting new technologies and developing precision medicine to provide personalized medical care. Government launching new programs or services to its residents. However there exist limited information on public perception and acceptance on this new branch of medicine. Therefore, this study attempts to understand the awareness and acceptance of the public on precision medicine. Also, we aim to determine the extent of utilization pattern of precision medicine services by the general population in the country.

## Methods

A population-based cross-sectional study was conducted using an online questionnaire. The questionnaire was distributed across online platforms. Participants were invited to access the questionnaire link after receiving a brief description of the study. Both Arabic and English versions of the questionnaire were made available through Google Forms. The study commenced only after receiving IRB approval (Ref. no. IRB-COP-STD-3-JULY-2024). Participation in the study was voluntary. An option was included in the survey link, allowing participants to share it on their social media platforms. Informed consent was provided for participants to accept before joining the study. UAE residents aged 18 years or older were eligible, and an eligibility check was implemented in the online tool.

The questionnaire, adapted from various studies and was modified in consultation with community and hospital pharmacists to align with the study objectives and the local context. It was initially prepared in English and then translated into Arabic using a forward-backward translation method to ensure linguistic and conceptual equivalence. A panel of five experts, including two university professors, two pharmacy students, and a community pharmacist, who were professionally familiar with the study objectives, reviewed the questionnaire for content and face validity. Later it was given for a common man to see the clarity and easiness to answer. Their feedback was incorporated to enhance clarity, relevance, and comprehensiveness. The final questionnaire consisted of 21 items divided into five sections: The sections included five questions about demographics, three questions about current health, three questions about knowledge of precision medicine, two questions about acceptance of precision medicine, and eight questions about the utilization patterns and practices of precision medicine services locally. Participants were given the option to complete the questionnaire in either Arabic or English to accommodate language preferences and improve response rates. 

Descriptive statistics were presented as mean ± standard deviation (SD) for continuous variables, and as frequencies and percentages for categorical variables. The levels of Knowledge, Awareness, and Acceptance of Precision Medicine were assessed. Comparisons of continuous demographic and clinical variables with Knowledge, Awareness, and Acceptance scores were conducted using the Mann–Whitney U test, as the data were not normally distributed. A p-value < 0.05 was considered statistically significant. All statistical analyses were performed using IBM SPSS Statistics for Windows, Version 26.0 (IBM Corp., Armonk, NY, USA).

## Results

A total of 402 individuals participated in the survey, with 400 completing it after excluding responses with missing data. Majority [n = 377(94.3%)] of our participants were young and educated above the high school level [n = 282] with most respondents holding a bachelor’s degree (60.0%). Income distribution varies, with 30.8% earning between 5,000–30,000 AED per month, while 26.5% preferred not to disclose their income. A total of 15.0% (n = 60) of participants indicated that they had one or more chronic health conditions, such as diabetes, hypertension and Asthma. Of these participants, 18.3% (n = 73), indicated they took one medication daily. The detail of demographic is presented in Table 1.

**Table 1.** Demographic details of the study participants.

Variables	n	%
**Gender**		
Female	256	64.0%
Male	144	36.0%
**Age**		
<50 Years	377	94.3%
>=50 Years	23	5.8%
**Education**		
Primary degree	12	3.0%
High school degree	106	26.5%
Undergraduate/Bachelor degree	240	60.0%
Post graduate/Master	37	9.3%
Doctorate	5	1.3%
**Occupation**		
Unemployed	31	7.8%
Student	191	47.8%
Employed	152	38.0%
Retired/Stay at home parent	26	6.5%
**Annual household income per month**		
Less than 5,000 AED	52	13.0%
More than 5,000 to less than 30,000 AED	123	30.8%
More than 30,000 to less than 60,000 AED	84	21.0%
Above 60,000 AED	35	8.8%
Prefer not to say	106	26.5%
**Chronic Diseases**		
No	340	85.0%
Yes	60	15.0%
**Medications/day**		
>5	6	1.5%
0	276	69.0%
1	73	18.3%
2 to 4	45	11.3%

### Knowledge and familiarity with key terminologies in precision medicine

**Figure 1.**
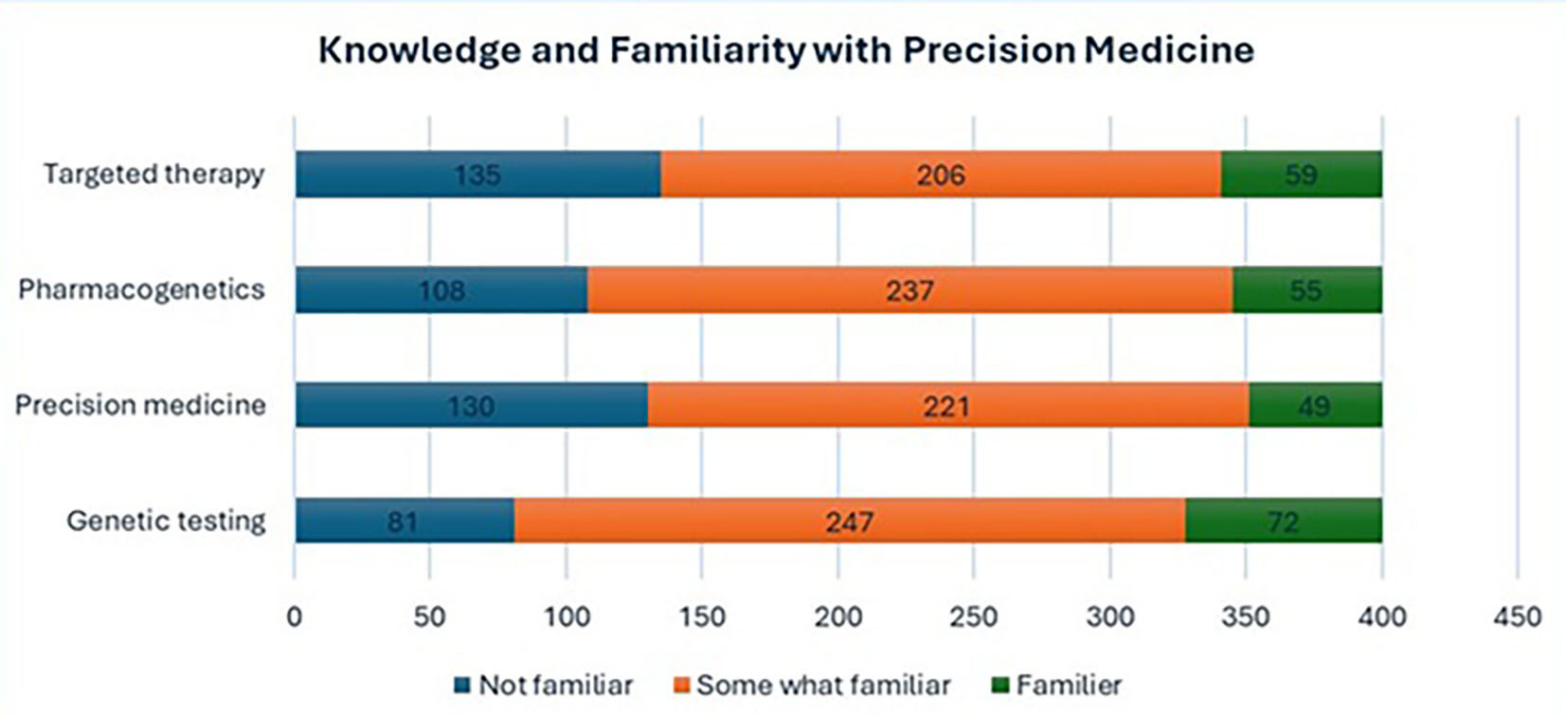
Participants' familiarity with key precision medicine terminologies.

The majority were slightly or somewhat familiar with terms such as “genetic testing” (61.8%), “precision medicine” (55.3%), “pharmacogenetics” (59.3%), and “targeted therapy” (51.5%). However, a notable proportion of participants reported being not familiar with these terms, ranging from 20.3% to 33.8%. Only a small percentage (12.3%–18.0%) indicated moderate or extreme familiarity. Details Participants’ familiarity with key precision medicine terminologies given in Figure 1.

### Sources of information on precision medicine

**Figure 2.**
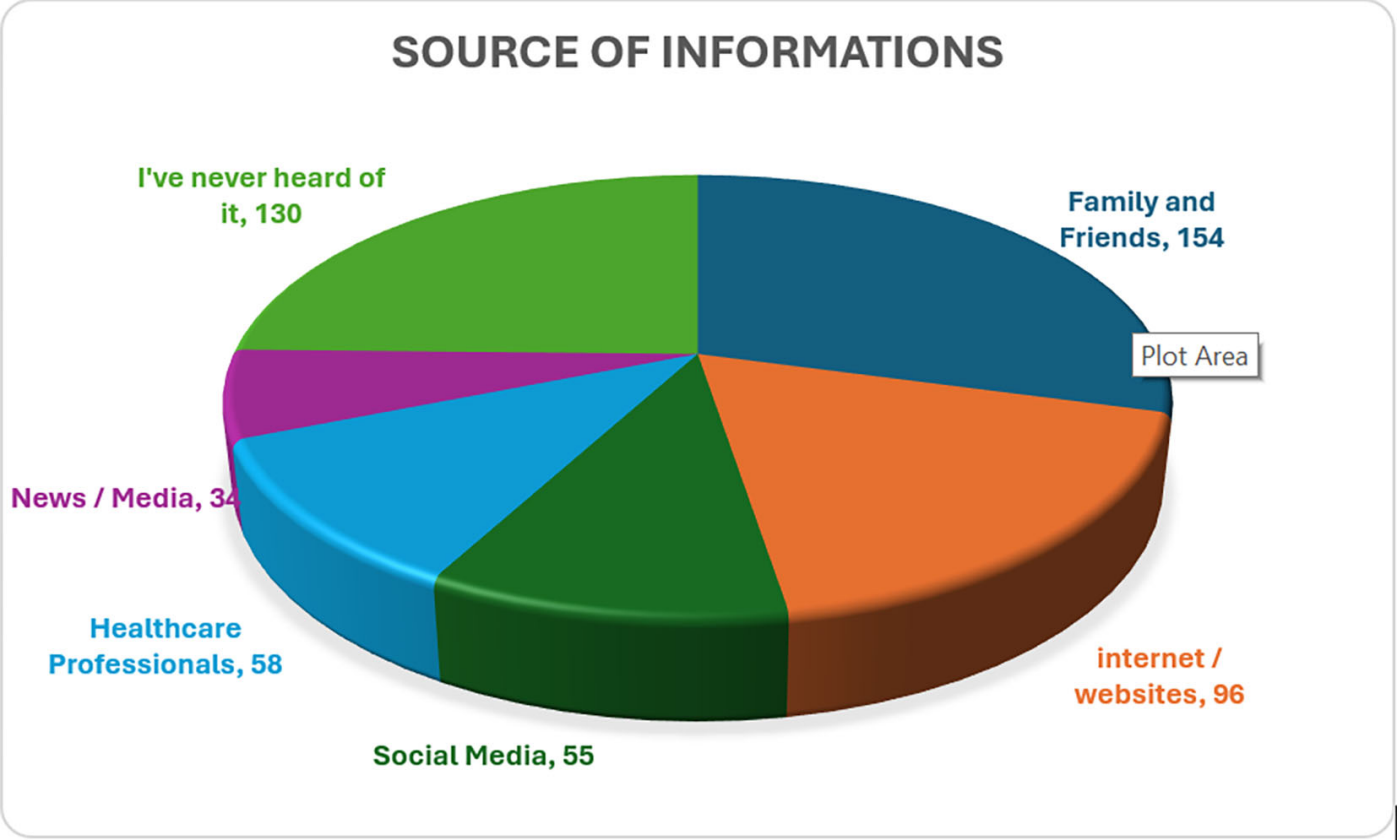
Sources of information on precision medicine.

Most participants (n = 154, 38.5%) identified family and friends as their primary source of information about precision medicine, followed by the internet and websites (n = 96, 24%). In contrast, a notable proportion of participants (n = 130, 32.5%) stated that they had never heard of precision medicine. Details is elicited in Figure 2.

### Perceived benefits of precision medicine

**Figure 3.**
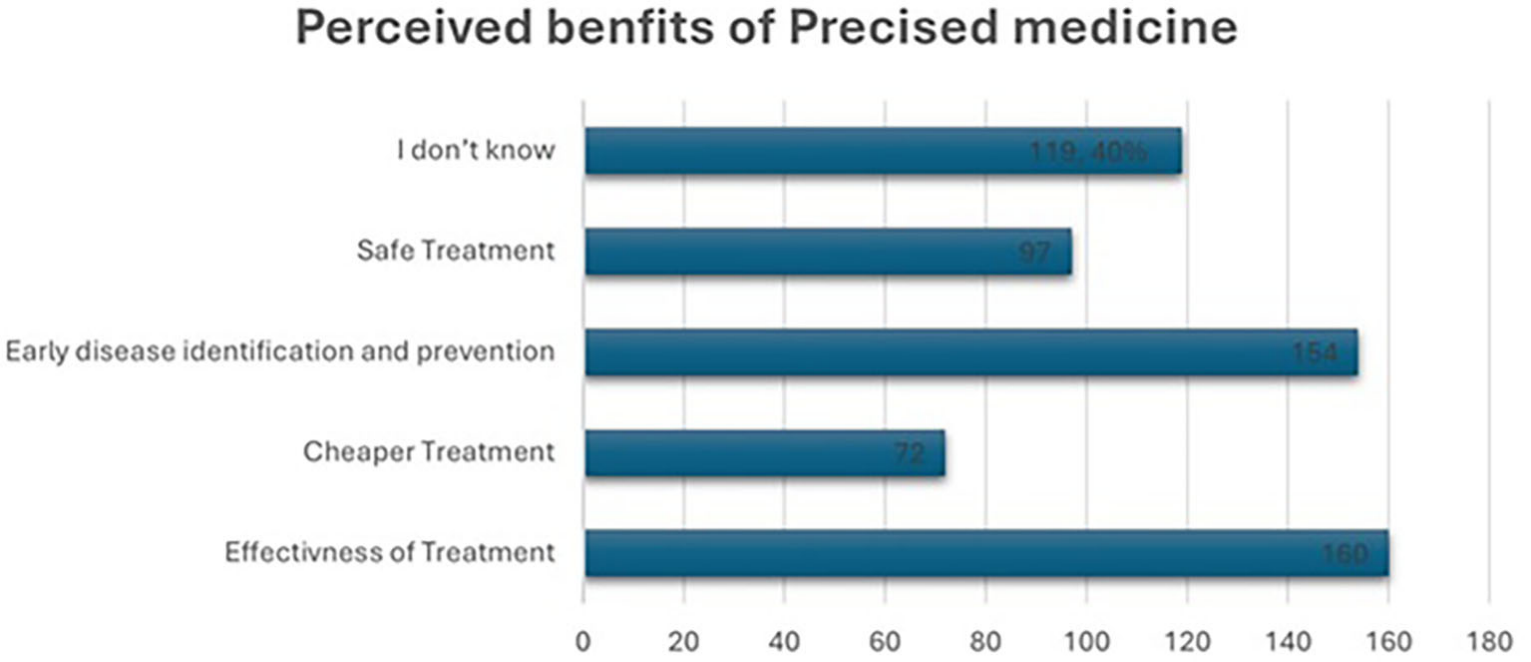
Perceived benefits of precision medicine.

As shown in the Figure 3, 40% of participants considered precision medicine main benefit is to be effective treatment, while 38.5% believed it could play a crucial role in early disease identification and prevention. For 18% of respondents, the key purpose was cheaper treatment, and 24.3% felt it was valuable for safe treatment. However, 29.8% of people were unsure about the specific benefits, suggesting that there’s a need for more education and understanding about how pharmacogenomic testing can be used to improve healthcare.

### Perceptions of insurance coverage for precision medicine among participants

**Figure 4.**
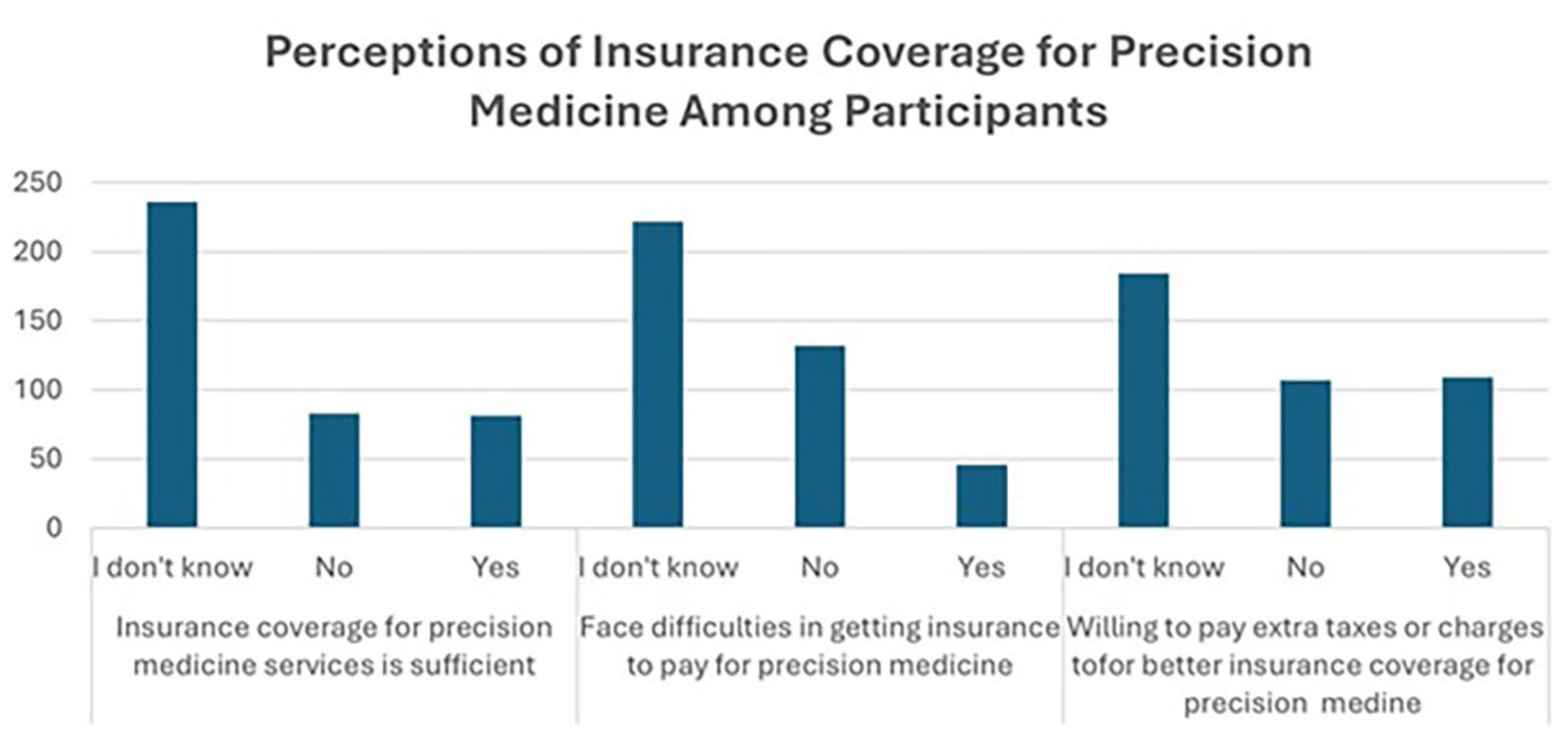
Perceptions of insurance coverage for precision medicine among participants.

Figure 4, illustrate the limited awareness and uncertainty regarding insurance coverage for precision medicine. A majority (59.0%) are unsure if their insurance is sufficient, while 20.8% believe it is inadequate, and only 20.3% think it is sufficient. When asked about challenges in obtaining insurance coverage for precision medicine treatments, 55.5% are uncertain, 33.0% have not faced difficulties, while 11.5% have encountered issues, indicating potential barriers to access.

### Exploring participants’ engagement in genetic testing

The data shows that the majority of participants have limited experience or engagement with genetic testing and precision medicine. For prior experience with genetic testing, 56.0% reported no experience, When it comes to discussions with doctors about genetic testing or medication, 55.5% of participants had not had such discussions, Regarding the utilization of precision medicine services by their families, 48.0% had not used such services, 36.8% were uncertain, and only 15.3% reported having utilized these services. Additionally, 44.8% of participants were unsure about their engagement with information on precision medicine and genetic testing, 36.8% had not engaged with any information, and 18.5% had actively sought such information. These findings suggest that awareness and engagement with these advanced medical fields remain limited, with a need for further education and outreach. ( Table 2)

**Table 2.** Prior experience with genetic testing.

Prior Experience with Genetic Testing	I don’t know	123	30.8%
	No	224	56.0%
	Yes	53	13.3%
Prior Discussions with Doctors About genetic testing or medication	I don’t know	119	29.8%
	No	222	55.5%
	Yes	59	14.8%
Utilization of Precision Medicine Services by Their Families	I don’t know	147	36.8%
	No	192	48.0%
	Yes	61	15.3%
Engagement with Information on Precision Medicine and Genetic Testing	I don’t know	179	44.8%
	No	147	36.8%
	Yes	74	18.5%

### Demographic variables vs knowledge

Table 3 explores variations of familiarity of words genetic testing, precision medicine, pharmacogenetics and targeted therapyacross demographic and socioeconomic groups in relation to precision medicine. Familiarity of words considered as the total score for each of words coded as “Not familiar”:1, “Slightly familiar”:2, “Somewhat familiar”:3, “Moderately familiar”:4, and “Extremely familiar”:5. The total score ranges from 4 to 20. A higher score indicates greater familiarity with these concepts.

**Table 3.** Demographic influences on knowledge about precision medicine.

Variables	Knowledge about precision medicine [Median (IQR)]	P value
**Gender**		
Female	8 (7,11)	0.056 *
Male	8 (6,11)	
**Age**		
<=50 Years	8 (7,11)	0.468 *
>50 Years	8 (6,10)	
**Education**		
Primary degree	8 (7,8)	0.431 ^#^
High school degree	8 (7,10)	
Undergraduate/Bachelor degree	8 (7,12)	
Post graduate/Master	8 (7,12)	
Doctorate	6 (5,9)	
**Occupation**		
Unemployed	8 (6,12)	<0.001 ^#^
Student	9 (8,12)	
Employed	8 (6,9.5)	
Stay at home parent/Retired	7.5 (6,9)	
**Annual household income per month**		
Less than 5,000 AED	8 (5,10)	0.004 ^#^
More than 5,000 to less than 30,000 AED	8 (7,11)	
More than 30,000 to less than 60,000 AED	8 (7,8)	
Above 60,000 AED	8 (7,12)	
Prefer not to say	10 (7,12)	
**Chronic Diseases**		
No	8(6,11)	0.414 *
Yes	8(7,11.5)	

Note:

* Indicates p value from Man-Whitney U test.

^#^ Indicates p value from Kruskal Wallis Test.

Gender differences approached statistical significance (p = 0.056), with females having a slightly higher median familiarity score (8, IQR: 7,11) compared to males (8, IQR: 6,11), suggesting a potential trend of greater awareness among females. Age and Education level did not show a significant association (p < 0.5), Occupation showed a strong association with familiarity (p < 0.001), with students having the highest median score (9, IQR: 8,12), indicating greater awareness, possibly due to academic exposure. Annual household income was significantly associated with familiarity (p = 0.004), with higher-income individuals (>60,000 AED) and those who preferred not to disclose their income showing the highest familiarity scores (10, IQR: 7,12). Those in the lowest income category (<5,000 AED) had a lower median familiarity (8, IQR, 5,10), indicating that economic factors may influence awareness levels.

### Demographic variables vs acceptances of precision medicine

Table 4 evaluates the acceptance of precision medicine based on a combined score derived from perceptions of healthcare outcome improvements and preferences for pharmacogenetic testing. The median score across all groups is generally around 5, indicating a neutral to slightly positive stance toward precision medicine. Overall, gender, age, education, occupation, and income do not show strong associations with acceptance. While chronic health conditions shows a statistically significant association with acceptance (p = 0.004). Individuals with chronic illnesses have a higher median score (6, IQR: 5,7) compared to those without (5, IQR: 4,6). This suggests that those managing chronic diseases may be more receptive to precision medicine, potentially due to a greater perceived need for personalized treatment options.

**Table 4.** Demographic influences on acceptances of precision medicine.

	Acceptance of precision medicine [Median (IQR)]	P value
**Gender**		
Female	5 (4,6)	0.975 *
Male	5 (4,6)	
**Age**		
<=50 Years	5 (4,6)	0.490 *
>50 Years	5 (5,6)	
**Education**		
Primary degree	5 (3.5,6)	0.339 ^#^
High school degree	5 (4,6)	
Undergraduate/Bachelor degree	5 (4,6)	
Post graduate/Master	6 (4,6)	
Doctorate	5 (4,5)	
**Occupation**		
Unemployed	4 (3,6)	0.170 ^#^
Student	5 (4,6)	
Employed	5 (4,6)	
Stay at home parent/Retired	5 (4,6)	
**Annual household income per month**		
Less than 5,000 AED	5 (4,7)	0.237 ^#^
More than 5,000 to less than 30,000 AED	5 (4,6)	
More than 30,000 to less than 60,000 AED	5 (4,6)	
Above 60,000 AED	5 (4,6)	
Prefer not to say	5 (4,6)	
**Chronic diseases**		
No	5 (4,6)	0.004 *
Yes	6 (5,7)	

Note:

* Indicates p value from Man-Whitney U test.

^#^ Indicates p value from Kruskal Wallis Test.

### Demographic vs practice of precision medicine

The Table 5 reports the variation in practice of precision medicine scores across different demographic and socioeconomic groups. A total score was calculated by summing the variables of have you ever done genetic testing before, have you ever discussed with precision medicine to doctor before and Did you or family members ever use precision medicine by coded as “I don’t know:0”, “No:1” and “Yes:2”. Total Score have a minimum value of 0 and maximum of 6 generated. Gender showed a statistically significant difference (p = 0.003), with females having a median score of 3 (IQR: 0,3) compared to males with a slightly higher range (3, IQR: 2,4), suggesting that males may have a broader practice distribution. Occupation, age and education was not significantly associated with practice scores. However, annual household income showed a highly significant association (p < 0.001), and detailed analysis indicates that individuals with an annual income between 30,000 and 60,000 AED (0, IQR: 0, 3) had lower levels of practice regarding precision medicine compared to those earning less than 5,000 AED (3, IQR: 2.5,3) and between 5,000 and 30,000 AED (3, IQR: 0,4). Lastly, chronic health conditions were significantly associated with awareness (p = 0.023), as individuals with such conditions had a higher median (3, IQR: 2,4) compared to those without (3, IQR: 0,3), implying that personal health experiences may contribute to greater awareness of precision medicine.

**Table 5.** Demographic influences on practices of precision medicine.

	Practices of precision medicine [Median (IQR)]	P value
**Gender**		
Female	3 (0,3)	0.003 *
Male	3 (2,4)	
**Age**		
<=50 Years	3 (0,3)	0.321 *
>50 Years	3 (2,3)	
**Education**		
Primary degree	3 (0.5,4.5)	0.052 ^#^
High school degree	3 (0,3)	
Undergraduate/Bachelor degree	3 (2,3)	
Post graduate/Master	3 (2,4)	
Doctorate	2 (2,3)	
**Occupation**		
Unemployed	3 (0,3)	0.332 ^#^
Student	3 (2,3)	
Employed	3 (0,4)	
Stay at home parent/Retired	3 (2,3)	
**Annual household income per month**		
Less than 5,000 AED	3 (2.5,3)	<0.001 ^#^
More than 5,000 to less than 30,000 AED	3 (1,3)	
More than 30,000 to less than 60,000 AED	0 (0,3)	
Above 60,000 AED	3 (0,4)	
Prefer not to say	3 (2,3)	
**Chronic disease**		
No	3 (0,3)	0.023 *
Yes	3 (2,4)	

Note:

* Indicates p value from Man-Whitney U test.

^#^ Indicates p value from Kruskal Wallis Test.

## Discussion

This study highlights the public’s awareness, acceptance, and utilization of precision medicine services in the UAE, a country at the forefront of adopting innovative healthcare technologies. The majority of participants were young, with 94.3% aged below 50 years, aligning with the UAE’s population demographics where younger adults constitute a significant portion of the workforce. ^12^ Additionally, the survey observed a higher proportion of female respondents (64.0%) compared to males (36.0%). Similar gender biases have been noted in health-related surveys globally, where women often exhibit higher participation due to increased engagement in healthcare matters. ^13, 14^ The survey highlights a highly educated population, with 60% holding undergraduate degrees, and 1.3% having completed doctoral studies. This educational profile is indicative of a population capable of understanding and adopting novel healthcare approaches, such as precision medicine. Among the surveyed participants, 15.0% reported having at least one chronic condition such as diabetes, hypertension, or asthma. This prevalence aligns with national statistics showing a growing burden of chronic diseases in the UAE due to lifestyle factors like sedentary behaviour and dietary habits. ^15^ Medication use patterns indicate that 18.3% of participants take one medication daily, and only 1.5% reported taking more than five medications. These findings are consistent with studies highlighting polypharmacy trends in older populations, with relatively lower rates among younger demographics. ^16^

The findings of this study reveal that while participants demonstrated slight familiarity with certain precision medicine terminologies, a significant proportion remained unfamiliar with these terms. This highlights the need for targeted public education and awareness campaigns to address the knowledge gap in precision medicine. In contrast, a study conducted by Edris et al. reported that participants exhibited a positive attitude toward precision medicine and pharmacogenomics research. ^17^

Similarly, most participants (38.5%) learned about precision medicine through family and friends, emphasizing the role of informal communication in disseminating knowledge. However, in contrast to these findings, Almaazmi et al. ^18^ reported that social media and family and friends had lower trustworthiness scores in regards to provide the medical information’s. The survey results revealed a positive outlook toward the potential benefits of pharmacogenomic testing, with 40% of participants recognizing its value in optimizing drug effectiveness, and 38.5% highlighting its role in preventing adverse drug reactions. These findings align with the growing body of literature that emphasizes pharmacogenomics’ potential to enhance medication efficacy and reduce adverse outcomes by tailoring treatments to individuals’ genetic profiles. ^17^

The exist a limited awareness surrounding insurance coverage for precision medicine among participants. In this study, 59% of participants were unsure about their insurance coverage for precision medicine, These results are consistent with research by Schroll MM et al. ^19^ who found that insurance coverage and reimbursement were significant barriers to the adoption of precision medicine, particularly for treatments involving genetic testing like cancer.

## Conclusion

This study highlights a moderate level of awareness and a generally positive perception of precision medicine among UAE residents. Participants recognized the potential benefits of pharmacogenomics. However, there remains a considerable gap in public understanding, these findings underscore the need for targeted public education campaigns, clearer communication from healthcare providers, and improved access to precision medicine services. Future research should focus on identifying barriers to adoption and developing strategies for broader implementation of precision medicine in healthcare.

## Ethics approval statement

This study was reviewed and approved by the Institutional Review Board (IRB) of Gulf Medical University, Ajman, United Arab Emirates (IRB Reference No: IRB-COP-STD-3-JULY-2024). This study involving human participants were conducted in accordance with the ethical standards of the institutional research committee and with Declaration of Helsinki.

## Consent statement

Written informed consent was obtained electronically through online checkpoints, where participants indicated their agreement by clicking on confirmatory statements prior to survey completion. Participant data were anonymized to ensure confidentiality and compliance with ethical research guidelines.

## Data Availability

Harvard Dataverse “Public awareness on precision medicine”, https://doi.org/10.7910/DVN/MNLFUY ^20^ Data are available under the terms of the Creative Commons CC0 1.0 Universal Public Domain Dedication (CC0 1.0).
