# Personalized Care in the UAE : A Study on Precision Medicine Awareness and Accessibility among the general population

**DOI:** 10.12688/f1000research.163687.2

**Published:** 2026-01-24

**Authors:** Jisha Myalil Lucca, Alhammadi Salama, Basant Abdalla, Jeswin Baby

**Affiliations:** 1 College of pharmacy, Gulf medical university, Ajman, United Arab Emirates; 2 Caritas Hospital & Institute of Health Sciences, Kottayam, Kerala, India

**Keywords:** Precision Medicine, Pharmacogenomics, Genetic Testing, Public Awareness, UAE

## Abstract

**Background:**

Precision medicine is an emerging approach that tailors treatments based on an individual’s genetic profile. The UAE has made significant strides in this field through initiatives like the National Genomics Strategy and the Emirati Genome Program. However, public awareness and engagement remain key challenges.

**Purpose:**

This study assesses awareness, acceptance, and utilization of precision medicine among UAE residents.

**Methods:**

A cross-sectional online survey was conducted using a snowball sampling method. The survey collected demographic data, health status, knowledge, and experiences with precision medicine. Descriptive statistics and chi-square tests were used to analyze associations.

**Results:**

Most participants (94.3%) were under 50 years old, 62.5% were female, and 60.0% held a bachelor’s degree. Awareness of precision medicine was moderate (55.3%), with higher familiarity among females and students. While 40% believed its main benefit was optimizing drug effectiveness, 38.5% viewed it as crucial for preventing adverse drug reactions. Family and friends (29.5%) were the primary sources of information, yet 25.5% had never heard of precision medicine. Awareness of insurance coverage was low, with 59.0% uncertain about their policy. Genetic testing participation was associated with education level (p < 0.05). Acceptance of precision medicine was higher among individuals with chronic illnesses (p = 0.004). Familiarity scores varied significantly by occupation (p < 0.001) and income (p = 0.004), with higher-income individuals showing greater awareness. Males had a broader range of practice scores (p = 0.003), and individuals with chronic conditions were more aware of precision medicine (p = 0.023).

**Conclusion:**

Despite advancements, public engagement with precision medicine remains limited. Targeted educational initiatives, improved accessibility, and increased awareness of insurance coverage may enhance adoption and utilization.

## Introduction

Precision medicine, a novel approach in healthcare, provides an optimized, targeted treatment to individual patients based on genetic composition, environmental factors, and lifestyle. ^1, 2^ It represents a revolutionary change in the treatment of rare and difficult to treat diseases by replacing the conventional one-size-fits-all treatment approach. ^3^ According to FDA “Personalized medicine, sometimes called individualized or precision medicine, is an evolving field in which physicians use diagnostic tests to determine which medical treatments will work best for each patient or use medical interventions to alter molecular mechanisms that impact health. By combining data from diagnostic tests with an individual’s medical history, circumstances, and values, healthcare providers can develop targeted treatment and prevention plans with their patients.” ^4^ The main aim of precision medicine is to pair the treatment with unique characteristics of each patient and to enhance the safety and efficacy of medications. Beyond cancer treatment, precision medicine impacts extend in treating asthma, infectious diseases, connective tissue diseases, cardiovascular diseases, obesity and diabetes. ^2, 3, 5, 6^ Precision medicine have been shown to decrease the healthcare expenses and minimize physicians’ burden, foster more personalized patient care. ^7^ According to the FDA precision medicine report in 2022 a total of 12 new drugs identified as personalized medicines and 5 new gene or cell-based therapies were approved. ^4^

The healthcare system in the Middle East is progressively advancing toward the integration of precision and genomic medicine into clinical practice. By implementing genetic medicine, the Middle East can prioritize the treatment of high prevalence chronic diseases, rare genetic and orphan disorders in the region. ^8^ Precision medicine market in the Middle East and Africa is set to expand at CAGR of 9.96 percent to a revenue worth of US$2.51 billion by 2023. ^9^ From 2023 to 2028, the precision medicine market in the MEA region is expected to expand at an average CAGR of 11.32%. Due to the market’s size, it is expected to increase from USD 4.98 billion in 2023 to USD 8.52 billion by 2028. ^9^

In March 2023, UAE launched a national genomics strategy to enhance the advancement of preventive medicine in the country. The Emirati Genome Program is a foundational project within the National Genome Strategy, which aims to use genomic data to enhance public health among UAE nationals. ^10, 11^ The first Personalized Precision Medicine Program for oncology was launched by the Abu Dhabi Department of Health. ^10, 11^ The main goal of the program is to provide healthcare professionals with high-quality information that will enable them to offer advanced diagnostics, customized treatment options, and prevention programs that are tailored to each individual’s unique genetic composition. ^10, 11^ It will also help in the development of innovative treatments for uncommon and chronic illnesses as well as improved prediction and prevention of current and future genetic diseases.

Recent research in the UAE and globally underscores both positive receptivity to precision medicine and significant gaps in awareness, capacity, and infrastructure that continue to limit accessibility. For example, a national survey among community pharmacists in the UAE reported moderate knowledge levels, with a mean score of 75.1%, highlighting the need for further education and training in this area. ^12^ Similarly, a study among medical and health sciences students found that knowledge of pharmacogenomics was generally fair and strongly influenced by factors such as year of study and prior exposure to formal training programs. ^13, 14^ Broader surveys across healthcare workers in the UAE have also revealed that, despite recognizing the potential benefits of genetic testing and personalized therapies, many still encounter barriers including cost, lack of provider education, and concerns around ethical and privacy issues. ^14^ These findings emphasize that while the foundation for precision medicine in the UAE is promising, considerable efforts are still required to strengthen knowledge, address structural barriers, and ensure equitable access to its benefits.

Recent healthcare initiatives in the UAE reflect an increasing focus on the adoption of advanced technologies and the integration of precision medicine to enhance individualized treatment approaches. The government has launched a new program and services to its residents. However, there is limited information on public perception and acceptance of this new branch of medicine.

Therefore, this study aims to understand the awareness and acceptance of the public on precision medicine. Additionally, we aim to determine the extent of utilization pattern of precision medicine services among the general population in the country.

## Methods

### Study design and participants

A population-based cross-sectional study was conducted using an online questionnaire, distributed across multiple platforms, including university mailing lists, social media groups, and professional networks. Participants were invited to access the questionnaire link after receiving a brief description of the study. Both Arabic and English versions of the questionnaire were made available through Google Forms. The study commenced only after receiving IRB approval (Ref. no. IRB-COP-STD-3-JULY-2024). Participation was voluntary, and informed consent was obtained electronically prior to enrollment. UAE residents aged 18 years or older were eligible, and an eligibility check was implemented within the online tool. Participants were also given the option to share the questionnaire link on their social media platforms to increase reach.

### Questionnaire development and validation

The questionnaire was adapted from previous studies ^12, 15, 16^ and modified in consultation with community and hospital pharmacists to ensure alignment with the study objectives and the local context. It was initially prepared in English and translated into Arabic using a forward–backward translation method to ensure linguistic and conceptual equivalence. A panel of five experts, including two university professors, two pharmacy students, and a community pharmacist familiar with the study objectives, reviewed the questionnaire for content and face validity. The instrument was further piloted with members of the general public to assess clarity, ease of understanding, and comprehensiveness, and feedback was incorporated accordingly. The final questionnaire consisted of 22 items across five sections: demographics (7 items), current health status (3 items), knowledge of precision medicine (2 items), acceptance of precision medicine (2 items), and utilization patterns of precision medicine services (8 items).

### Key constructs and scoring

Familiarity/knowledge of precision medicine was defined as participants’ self-reported awareness and understanding of core concepts, including genetic testing, pharmacogenetics, targeted therapy, and precision medicine itself. This was assessed using four Likert-scale items ranging from 1 (“Not familiar”) to 5 (“Extremely familiar”), and responses were summed to generate a total score ranging from 4 to 20, with higher scores indicating greater knowledge. Acceptance of precision medicine captured participants’ willingness to adopt personalized medicine approaches, measured using Likert-scale items (1 = strongly disagree to 5 = strongly agree) addressing perceived value, willingness to use, and comfort with precision medicine; summed item scores reflected overall acceptance. Perceived benefits represented participants’ recognition of advantages such as improved treatment effectiveness, reduced adverse outcomes, cost-effectiveness, and early disease detection, assessed via multiple-choice and Likert-scale items, with higher composite scores indicating stronger perceived benefits. Preferences for pharmacogenetic testing measured interest in undergoing testing, using Likert-scale items from 1 (“Not at all interested”) to 5 (“Extremely interested”), with higher scores reflecting greater preference. Finally, utilization or practice of precision medicine captured actual engagement, including prior genetic testing, discussions with healthcare providers, or use of precision medicine services; responses were coded as 0 (“I don’t know”), 1 (“No”), and 2 (“Yes”), and summed across items to generate a total score (range 0–6), with higher scores indicating more active engagement.

To minimize duplicate responses, the Google Forms “one response per device” restriction was enabled, and entries with identical IP addresses or missing consent were excluded during data cleaning. Internal consistency was assessed to ensure reliability, with Cronbach’s alpha values of 0.852 for the knowledge section and 0.855 for the utilization section, indicating high reliability.

Scoring methods for knowledge, acceptance, and practice were developed based on previously published research on awareness and perceptions of precision medicine. ^17, 18^ Each domain comprised Likert-scale items, with composite scores generated by summing individual responses within each category. Higher scores reflected greater knowledge, higher acceptance, and more favorable practice behaviors toward precision medicine.

Participants were given the option to complete the questionnaire in either Arabic or English to accommodate language preferences and improve response rates. This comprehensive approach ensured the questionnaire’s validity, reliability, and data integrity before its distribution.

### Statistical analysis

Descriptive statistics for ordinal or non-normally distributed continuous variables are presented as medians with interquartile ranges (IQR), while categorical variables are reported as frequencies and percentages. The levels of Knowledge, Awareness, and Acceptance of Precision Medicine were assessed. Comparisons of continuous demographic and clinical variables with Knowledge, Awareness, and Acceptance scores were conducted using the Mann–Whitney U test, as the data were not normally distributed. A p-value < 0.05 was considered statistically significant. All statistical analyses were performed using IBM SPSS Statistics for Windows, Version 26.0 (IBM Corp., Armonk, NY, USA).

### Conceptional definitions

Precision Medicine (PM): The use of diagnostic tools and treatments targeted to the needs of the individual patient on the basis of genetic, biomarker, or psychosocial characteristics. PM does not imply the creation of medicines or devices that are unique to a patient, but rather the ability to stratify individuals into groups that differ in their susceptibility to a specific disease or their response to a specific treatment. ^1, 11^

Personalized Medicine: A synonym to precision medicine. However, “precision medicine” is the preferred term. ^1, 11^

Pharmacogenomics: The use of genomic information to inform prescription or avoidance of pharmaceuticals. ^1, 11^

## Results

A total of 402 individuals participated in the survey, with 400 completing it after excluding responses with missing data. The majority of participants were younger adults (n = 377, 94.3%) and had education levels above high school (n = 282, 70.5%), with most holding a bachelor’s degree (n = 240, 60.0%). Monthly household income varied with 123 participants (30.8%) earning between 5,000–30,000 AED, while 106 (26.5%) preferred not to disclose their income. A total of 60 participants (15.0%) reported having one or more chronic health conditions, such as diabetes, hypertension, or asthma. Among these, 73 participants (18.3%) reported taking at least one medication daily. Detailed demographic characteristics are presented in Table 1.

**Table 1.** Demographic details of the study participants.

Variables	n	%
**Gender**		
Female	256	64.0%
Male	144	36.0%
**Age**		
<50 Years	377	94.3%
>=50 Years	23	5.8%
**Education**		
Primary degree	12	3.0%
High school degree	106	26.5%
Undergraduate/Bachelor degree	240	60.0%
Post graduate/Master	37	9.3%
Doctorate	5	1.3%
**Occupation**		
Unemployed	31	7.8%
Student	191	47.8%
Employed	152	38.0%
Retired/Stay at home parent	26	6.5%
**Annual household income per month**		
Less than 5,000 AED	52	13.0%
More than 5,000 to less than 30,000 AED	123	30.8%
More than 30,000 to less than 60,000 AED	84	21.0%
Above 60,000 AED	35	8.8%
Prefer not to say	106	26.5%
**Chronic Diseases**		
No	340	85.0%
Yes	60	15.0%
**Medications/day**		
>5	6	1.5%
0	276	69.0%
1	73	18.3%
2 to 4	45	11.3%

### Knowledge and familiarity with key terminologies in precision medicine

**Figure 1.**
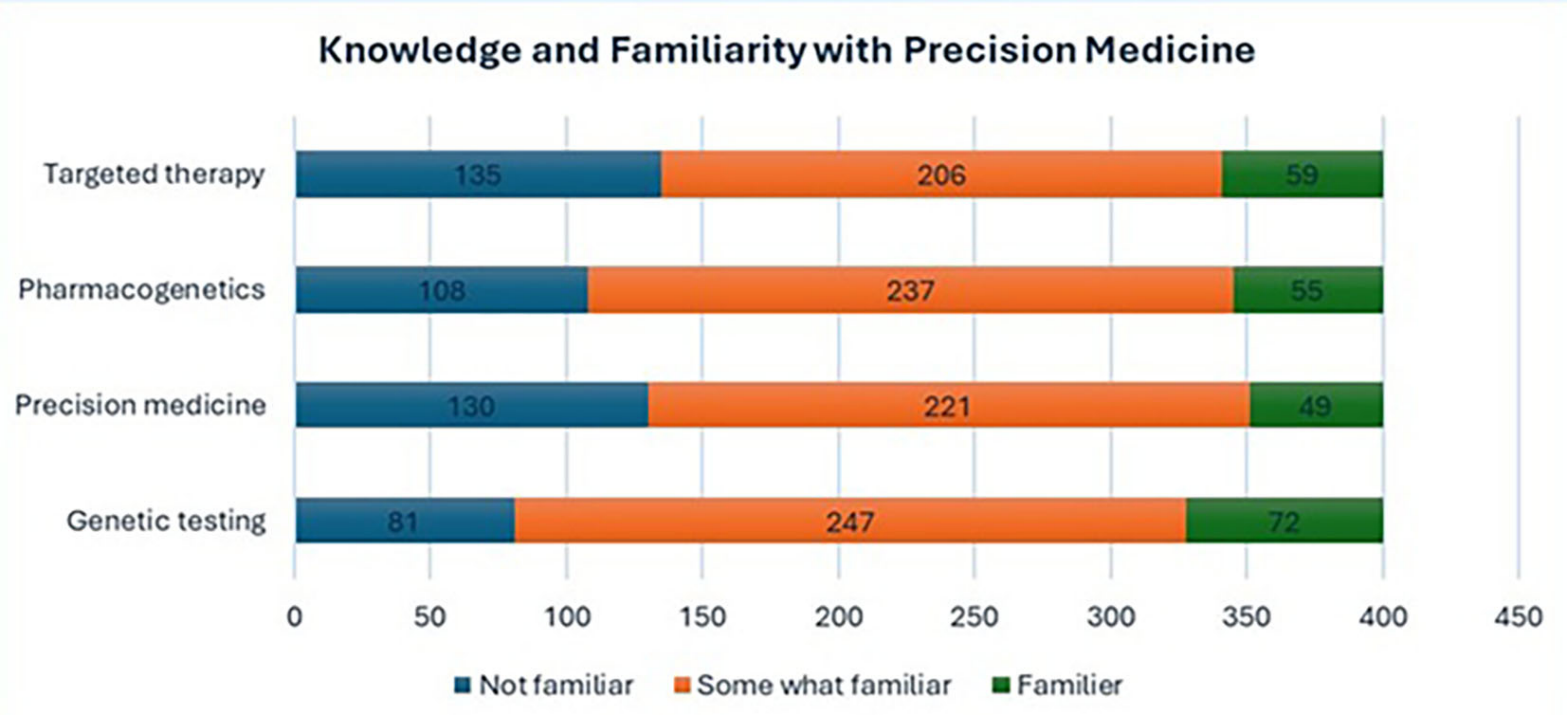
Participants' familiarity with key precision medicine terminologies.

The majority were slightly or somewhat familiar with terms such as “genetic testing” (61.8%), “precision medicine” (55.3%), “pharmacogenetics” (59.3%), and “targeted therapy” (51.5%). However, a notable proportion of participants reported being not familiar with these terms, ranging from 20.3% to 33.8%. Only a small percentage (12.3%–18.0%) indicated moderate or extreme familiarity. Details Participants’ familiarity with key precision medicine terminologies given in Figure 1.

### Sources of information on precision medicine

**Figure 2.**
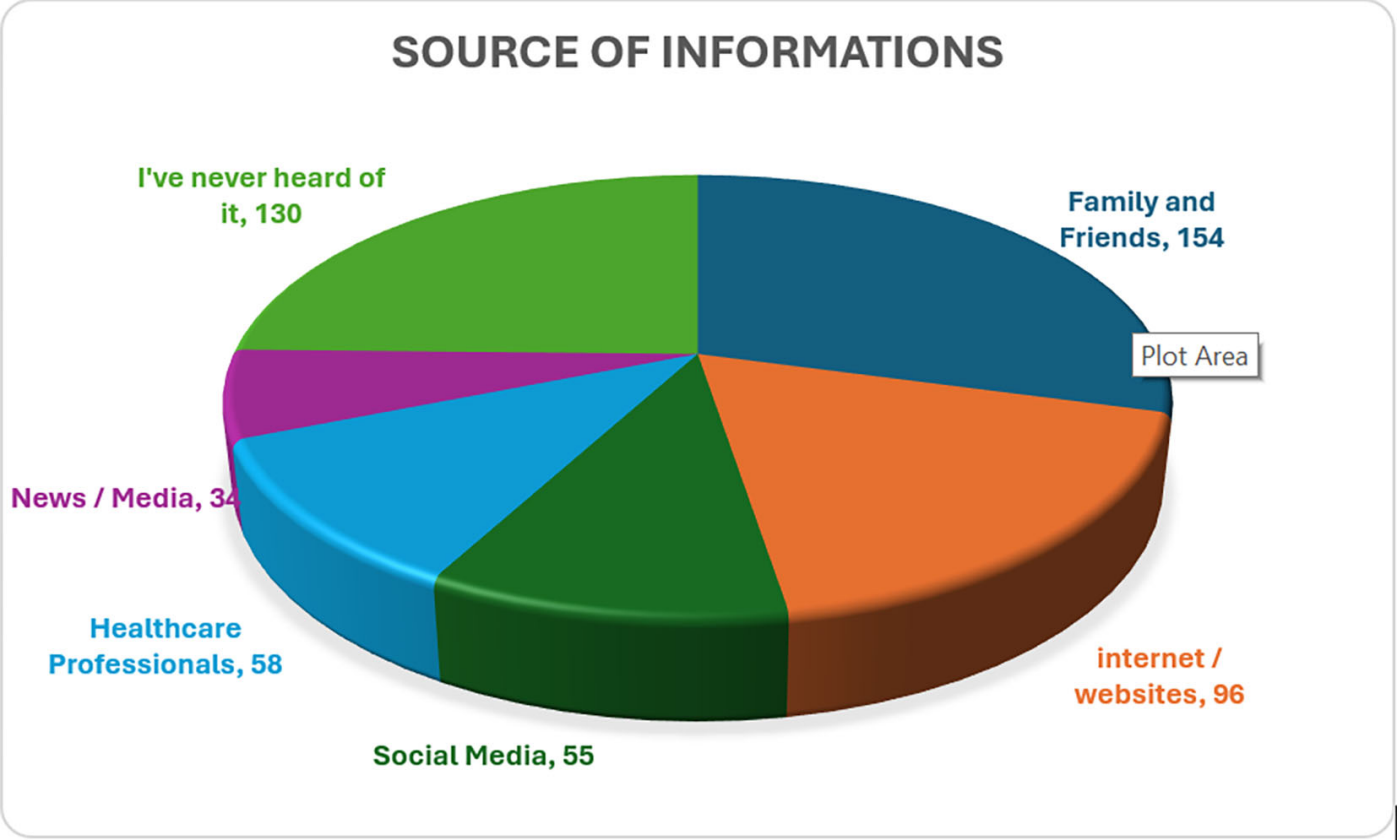
Sources of information on precision medicine.

Most participants (n = 154, 38.5%) identified family and friends as their primary source of information about precision medicine, followed by the internet and websites (n = 96, 24%). In contrast, a notable proportion of participants (n = 130, 32.5%) stated that they had never heard of precision medicine. Details are presented in Figure 2.

### Perceived benefits of precision medicine

**Figure 3.**
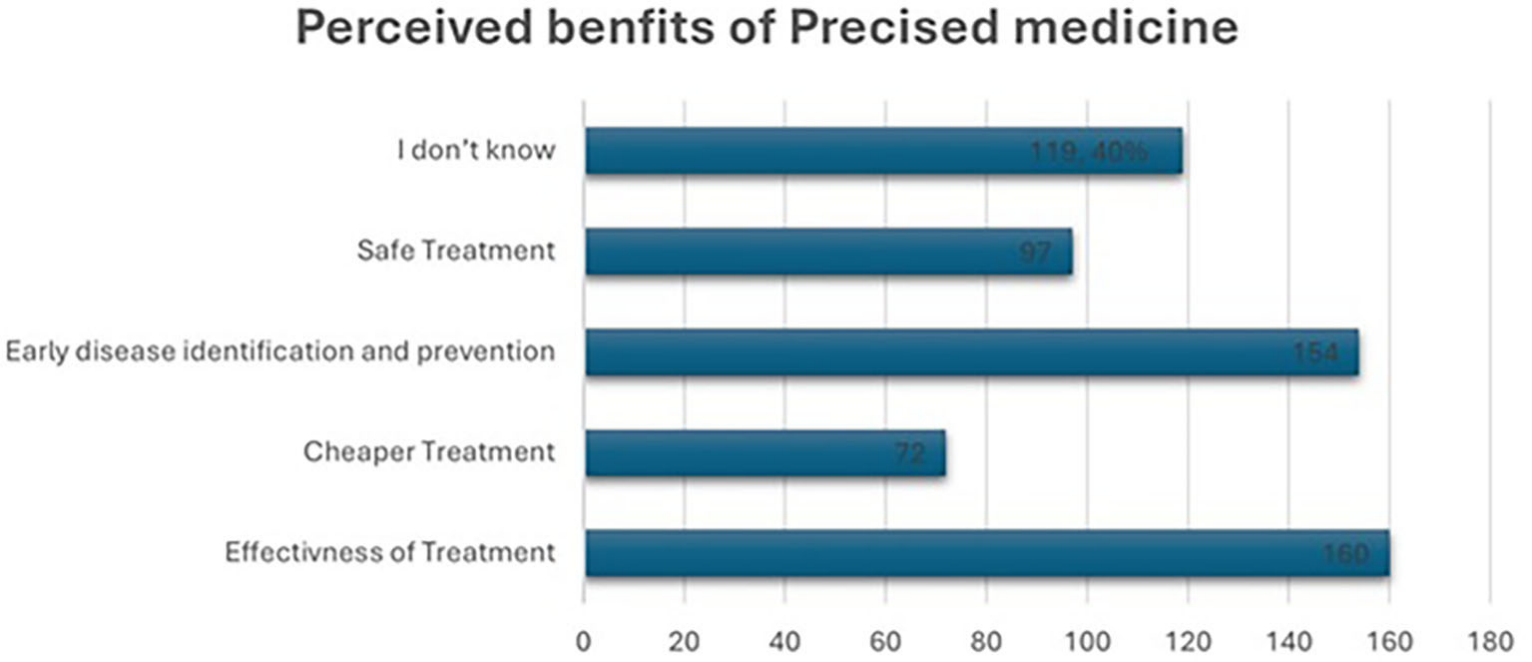
Perceived benefits of precision medicine.

As shown in Figure 3, 160 (40%) of participants considered precision medicine main benefit is to be effective treatment, while 154 (38.5%) believed it could play a crucial role in early disease identification and prevention. For 72 (18%) of respondents, the key purpose was cheaper treatment, and 97 (24.3%) reported that it was valuable for safe treatment. However, 119 (29.8%) of participants were unsure about the specific benefits.

### Perceptions of insurance coverage for precision medicine among participants

**Figure 4.**
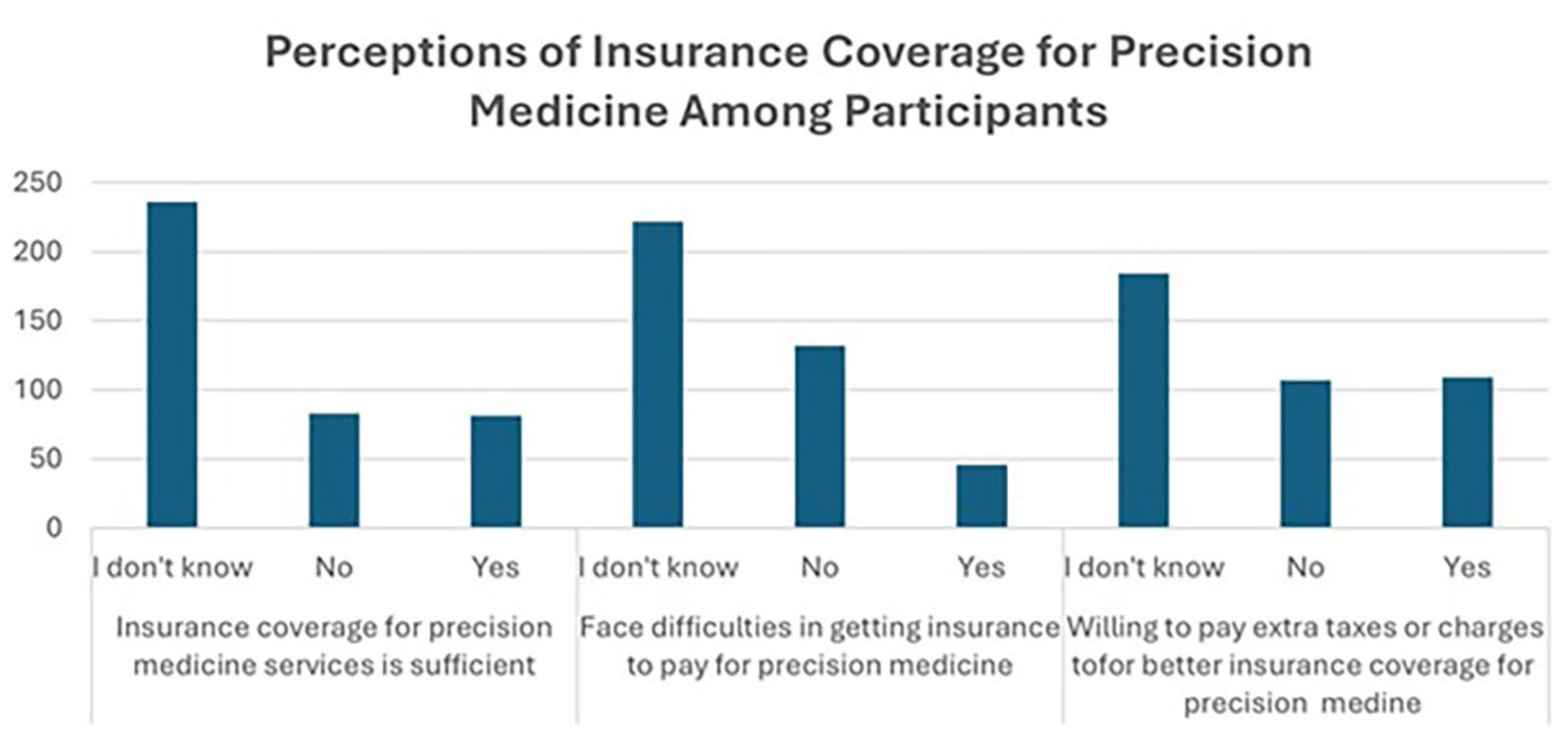
Perceptions of insurance coverage for precision medicine among participants.

Figure 4 illustrates the limited awareness and uncertainty regarding insurance coverage for precision medicine. A majority of participants, 236 (59.0%), were unsure if their insurance coverage was sufficient, while 83 (20.8%) believed it was inadequate, and 81 (20.3%) reported that it was sufficient. Regarding challenges in obtaining insurance coverage for precision medicine treatments, 222 (55.5%) participants were uncertain, 132 (33.0%) had not faced any difficulties, and 46 (11.5%) had encountered issues, indicating potential barriers to access.

### Exploring participants’ engagement in genetic testing

The data indicate that most participants have limited experience or engagement with genetic testing and precision medicine. Regarding prior experience with genetic testing, 224 (56.0%) reported having no experience. In terms of discussions with doctors about genetic testing or related treatments, 222 (55.5%) had not had such conversations. Concerning the utilization of precision medicine services by themselves or their families, 192 (48.0%) had not used such services, 147 (36.8%) were uncertain, and 61 (15.3%) reported having utilized these services. Additionally, 179 (44.8%) participants were unsure about their engagement with information on precision medicine and genetic testing, 147 (36.8%) had not engaged with any information, and 74 (18.5%) had actively sought such information. These findings suggest that public awareness and engagement with precision medicine remain limited, highlighting the need for targeted education and outreach initiatives ( Table 2).

**Table 2.** Prior experience with genetic testing.

Prior Experience with Genetic Testing	I don’t know	123	30.8%
	No	224	56.0%
	Yes	53	13.3%
Prior Discussions with Doctors About genetic testing or medication	I don’t know	119	29.8%
	No	222	55.5%
	Yes	59	14.8%
Utilization of Precision Medicine Services by Their Families	I don’t know	147	36.8%
	No	192	48.0%
	Yes	61	15.3%
Engagement with Information on Precision Medicine and Genetic Testing	I don’t know	179	44.8%
	No	147	36.8%
	Yes	74	18.5%

### Demographic variables vs knowledge

Table 3, Gender differences in familiarity scores were not statistically significant (p = 0.056), with both females and males showing a median score of 8 (IQR: females 7–11, males 6–11). Age and education level did not show a significant association (p =0.419 and p = 0.327, respectively), occupation showed a strong association with familiarity (p < 0.001), with students having the highest median score (9, IQR: 8,12), indicating greater awareness, possibly due to academic exposure. Annual household income was significantly associated with familiarity (p = 0.004), with higher-income individuals (>60,000 AED) and those who preferred not to disclose their income showing the highest familiarity scores (10, IQR: 7,12). Those in the lowest income category (<5,000 AED) had a lower median familiarity (8, IQR: 5,10), indicating that economic factors may influence awareness levels.

**Table 3.** Demographic influences on knowledge about precision medicine.

Variables	Knowledge about precision medicine [Median (IQR)]	P value
**Gender**		
Female	8 (7,11)	0.056 *
Male	8 (6,11)	
**Age**		
<=50 Years	8 (7,11)	0.468 *
>50 Years	8 (6,10)	
**Education**		
Primary degree	8 (7,8)	0.431 ^#^
High school degree	8 (7,10)	
Undergraduate/Bachelor degree	8 (7,12)	
Post graduate/Master	8 (7,12)	
Doctorate	6 (5,9)	
**Occupation**		
Unemployed	8 (6,12)	<0.001 ^#^
Student	9 (8,12)	
Employed	8 (6,9.5)	
Stay at home parent/Retired	7.5 (6,9)	
**Annual household income per month**		
Less than 5,000 AED	8 (5,10)	0.004 ^#^
More than 5,000 to less than 30,000 AED	8 (7,11)	
More than 30,000 to less than 60,000 AED	8 (7,8)	
Above 60,000 AED	8 (7,12)	
Prefer not to say	10 (7,12)	
**Chronic Diseases**		
No	8(6,11)	0.414 *
Yes	8(7,11.5)	

Note:

* Indicates p value from Man-Whitney U test.

^#^ Indicates p value from Kruskal Wallis Test.

### Demographic variables vs acceptances of precision medicine

Table 4 evaluates the acceptance of precision medicine based on a combined score derived from perceptions of healthcare outcome improvements and preferences for pharmacogenetic testing. The total acceptance score ranged from 1 to 5 for each item, with higher scores indicating greater acceptance. Across all demographic groups, the median acceptance score was **5 (neutral) on the 1–5 scale**, indicating a generally neutral to slightly positive stance toward precision medicine.

Overall, gender, age, education, occupation, and income did not show strong associations with acceptance, while chronic health conditions showed a statistically significant association with acceptance (p = 0.004). Individuals with chronic illnesses have a higher median score (6, IQR: 5,7) compared to those without (5, IQR: 4,6). This suggests that those managing chronic diseases may be more receptive to precision medicine, potentially due to a greater perceived need for personalized treatment options. This suggests that individuals managing chronic diseases may be more receptive to precision medicine, potentially due to a greater perceived need for personalized treatment options.

**Table 4.** Demographic influences on acceptances of precision medicine.

	Acceptance of precision medicine [Median (IQR)]	P value
**Gender**		
Female	5 (4,6)	0.975 *
Male	5 (4,6)	
**Age**		
<=50 Years	5 (4,6)	0.490 *
>50 Years	5 (5,6)	
**Education**		
Primary degree	5 (3.5,6)	0.339 ^#^
High school degree	5 (4,6)	
Undergraduate/Bachelor degree	5 (4,6)	
Post graduate/Master	6 (4,6)	
Doctorate	5 (4,5)	
**Occupation**		
Unemployed	4 (3,6)	0.170 ^#^
Student	5 (4,6)	
Employed	5 (4,6)	
Stay at home parent/Retired	5 (4,6)	
**Annual household income per month**		
Less than 5,000 AED	5 (4,7)	0.237 ^#^
More than 5,000 to less than 30,000 AED	5 (4,6)	
More than 30,000 to less than 60,000 AED	5 (4,6)	
Above 60,000 AED	5 (4,6)	
Prefer not to say	5 (4,6)	
**Chronic diseases**		
No	5 (4,6)	0.004 *
Yes	6 (5,7)	

Note:

* Indicates p value from Man-Whitney U test.

^#^ Indicates p value from Kruskal Wallis Test.

### Demographic vs practice of precision medicine

Table 5 reports the variation in practice of precision medicine scores across different demographic and socioeconomic groups. Gender showed a statistically significant difference (p = 0.003), with females having a median score of 3 (IQR: 0,3) compared to males with a slightly higher range (3, IQR: 2,4), suggesting that males may have a broader practice distribution. Occupation, age and education was not significantly associated with practice scores. However, annual household income showed a highly significant association (p < 0.001), and detailed analysis indicates that individuals with an annual income between 30,000 and 60,000 AED (0, IQR: 0, 3) had lower levels of practice regarding precision medicine compared to those earning less than 5,000 AED (3, IQR: 2.5,3) and between 5,000 and 30,000 AED (3, IQR: 0,4). Lastly, chronic health conditions were significantly associated with awareness (p = 0.023), as individuals with such conditions had a higher median (3, IQR: 2,4) compared to those without (3, IQR: 0,3), implying that personal health experiences may contribute to greater awareness of precision medicine.

**Table 5.** Demographic influences on practices of precision medicine.

	Practices of precision medicine [Median (IQR)]	P value
**Gender**		
Female	3 (0,3)	0.003 *
Male	3 (2,4)	
**Age**		
<=50 Years	3 (0,3)	0.321 *
>50 Years	3 (2,3)	
**Education**		
Primary degree	3 (0.5,4.5)	0.052 ^#^
High school degree	3 (0,3)	
Undergraduate/Bachelor degree	3 (2,3)	
Post graduate/Master	3 (2,4)	
Doctorate	2 (2,3)	
**Occupation**		
Unemployed	3 (0,3)	0.332 ^#^
Student	3 (2,3)	
Employed	3 (0,4)	
Stay at home parent/Retired	3 (2,3)	
**Annual household income per month**		
Less than 5,000 AED	3 (2.5,3)	<0.001 ^#^
More than 5,000 to less than 30,000 AED	3 (1,3)	
More than 30,000 to less than 60,000 AED	0 (0,3)	
Above 60,000 AED	3 (0,4)	
Prefer not to say	3 (2,3)	
**Chronic disease**		
No	3 (0,3)	0.023 *
Yes	3 (2,4)	

Note:

* Indicates p value from Man-Whitney U test.

^#^ Indicates p value from Kruskal Wallis Test.

## Discussion

This study highlights the public’s awareness, acceptance, and utilization of precision medicine services in the UAE, a country at the forefront of adopting innovative healthcare technologies. The majority of participants were young, with 94.3% aged below 50 years, aligning with the UAE’s population demographics where younger adults constitute a significant portion of the workforce. ^19^ Additionally, the survey observed a higher proportion of female respondents (64.0%) compared to males (36.0%). Similar gender biases have been noted in health-related surveys globally, where women often exhibit higher participation due to increased engagement in healthcare matters. ^20, 21^ The survey highlights a highly educated population, with 60% holding undergraduate degrees, and 1.3% having completed doctoral studies. This educational profile is indicative of a population capable of understanding and adopting novel healthcare approaches, such as precision medicine. Among the surveyed participants, 15.0% reported having at least one chronic condition such as diabetes, hypertension, or asthma. This prevalence aligns with national statistics showing a growing burden of chronic diseases in the UAE due to lifestyle factors like sedentary behaviour and dietary habits. ^17^ Medication use patterns indicate that 18.3% of participants take one medication daily, and only 1.5% reported taking more than five medications. These findings are consistent with studies highlighting polypharmacy trends in older populations, with relatively lower rates among younger demographics. ^18^

The findings of this study reveal that while participants demonstrated slight familiarity with certain precision medicine terminologies, a significant proportion remained unfamiliar with these terms. This highlights the need for targeted public education and awareness campaigns to address the knowledge gap in precision medicine. In contrast, a study conducted by Edris et al. reported that participants exhibited a positive attitude toward precision medicine and pharmacogenomics research. ^16^

Similarly, most participants (38.5%) learned about precision medicine through family and friends, emphasizing the role of informal communication in disseminating knowledge. However, in contrast to these findings, Almaazmi et al. ^22^ reported that social media and family and friends had lower trustworthiness scores in regards to provide the medical information’s. The survey results revealed a positive outlook toward the potential benefits of pharmacogenomic testing, with 40% of participants recognizing its value in optimizing drug effectiveness, and 38.5% highlighting its role in preventing adverse drug reactions. These findings align with the growing body of literature that emphasizes pharmacogenomics’ potential to enhance medication efficacy and reduce adverse outcomes by tailoring treatments to individuals’ genetic profiles. ^16^

There exists a significant level of uncertainty and limited awareness surrounding insurance coverage for precision medicine among participants. In this study, 59% of participants were unsure about their insurance coverage for precision medicine. These results are consistent with research by Schroll MM et al. ^23^ who found that insurance coverage and reimbursement were significant barriers to the adoption of precision medicine, particularly for treatments involving genetic testing like cancer.

## Limitations

This study has several limitations that should be acknowledged. First, the use of an online, snowball-sampling method may have introduced **selection bias**, resulting in an overrepresentation of younger, highly educated, and digitally active participants. Consequently, the findings may not be fully representative of the broader UAE population. Second, as the study relied on **self-reported data**, responses may be subject to recall and social desirability biases, potentially affecting the accuracy of reported awareness and practices.

Participants’ limited baseline awareness and engagement with precision medicine may have affected their understanding of items related to insurance coverage, potentially introducing measurement error and affecting the construct validity of these responses.

## Conclusion

This study provides exploratory insights into the current state of public awareness, acceptance, and utilization of precision medicine in the UAE. Overall, participants demonstrated limited to modest awareness and engagement in related practices. While some recognized potential benefits, particularly in improving treatment effectiveness and preventing adverse outcomes, substantial gaps remain in understanding the broader applications of precision medicine and its coverage under insurance. Participants with chronic health conditions demonstrated higher acceptance, suggesting a potential perceived need for personalized treatment options.

Given the exploratory nature and the non-representative sample, these findings should be interpreted cautiously. They highlight areas for further research, including evaluating healthcare providers’ perspectives, identifying barriers to adoption, and assessing the impact of public education or awareness initiatives. Such research can help inform strategies to enhance understanding, accessibility, and trust in precision medicine, although broader population-based studies are needed before drawing definitive conclusions.

## Ethics approval statement

This study was reviewed and approved by the Institutional Review Board (IRB) of Gulf Medical University, Ajman, United Arab Emirates (IRB Reference No: IRB-COP-STD-3-JULY-2024). This study involving human participants were conducted in accordance with the ethical standards of the institutional research committee and with Declaration of Helsinki.

## Consent statement

Written informed consent was obtained electronically through online checkpoints, where participants indicated their agreement by clicking on confirmatory statements prior to survey completion. Participant data were anonymized to ensure confidentiality and compliance with ethical research guidelines.

## Data Availability

Harvard Dataverse “Public awareness on precision medicine”, https://doi.org/10.7910/DVN/MNLFUY ^24^ Data are available under the terms of the Creative Commons CC0 1.0 Universal Public Domain Dedication (CC0 1.0).
