# Supplementary material for: Personalized Care in the UAE : A Study on Precision Medicine Awareness and Accessibility among the general population
Source: F1000Res. 2026 Jan 24;14:632. Originally published 2025 Jun 27. [Version 2] doi: 10.12688/f1000research.163687.2 (PMC12933041; doi:10.12688/f1000research.163687.2)
Supplement: Peer review history

# Reviewer report: Reviewer response for version 2

**DOI:** 10.5256/f1000research.191800.r452917

**Published:** 2026-02-24

**Authors:** Mohamed Zahir Alimohamed

**Affiliations:** 1 Muhimbili University of Health and Allied Sciences, Dar es Salaam, Tanzania

Additional comments:

- The limitations section is clearer now, but it would help to state more explicitly that the findings cannot be generalized to the entire UAE population, especially older adults and lower-education groups.
- The scoring systems for knowledge, acceptance, and practice are now explained, but a brief reminder in the Results section (one sentence) on what higher scores mean would improve readability for non-technical readers.
- In the Discussion, some comparisons with international studies are added. Consider briefly explaining why the UAE context may differ (health system structure, insurance model, population demographics) rather than only stating similarities or differences.
- The Conclusion is more balanced than before. One suggestion is to slightly reduce policy-forward language and keep recommendations framed as future research and public engagement priorities, not implementation guidance.
- Minor editorial point: ensure consistent tense and capitalization in table titles and figure captions. A final copy-edit will easily fix this.

Overall assessment:

 The revision addresses the core scientific and methodological issues. The manuscript now reads as a sound exploratory study, with appropriate caution in interpretation. Only minor refinements are needed at this stage.

Is the work clearly and accurately presented and does it cite the current literature?

Partly

If applicable, is the statistical analysis and its interpretation appropriate?

Yes

Are all the source data underlying the results available to ensure full reproducibility?

Yes

Is the study design appropriate and is the work technically sound?

Yes

Are the conclusions drawn adequately supported by the results?

Partly

Are sufficient details of methods and analysis provided to allow replication by others?

Partly

Reviewer Expertise:

Molecular Genetics, Genetic Counselling, Molecular diagnostics, Cardiomyopathies, SCD, NCDs, Precision Medicine.

I confirm that I have read this submission and believe that I have an appropriate level of expertise to confirm that it is of an acceptable scientific standard.

# Reviewer report: Reviewer response for version 2

**DOI:** 10.5256/f1000research.191800.r452919

**Published:** 2026-02-04

**Authors:** Colleen Aldous

**Affiliations:** 1 University of Kwa-Zulu Natal, Durban, South Africa

The article has been significantly improved and now it can be indexed.

Is the work clearly and accurately presented and does it cite the current literature?

Partly

If applicable, is the statistical analysis and its interpretation appropriate?

Partly

Are all the source data underlying the results available to ensure full reproducibility?

Partly

Is the study design appropriate and is the work technically sound?

Partly

Are the conclusions drawn adequately supported by the results?

Partly

Are sufficient details of methods and analysis provided to allow replication by others?

No

Reviewer Expertise:

NA

I confirm that I have read this submission and believe that I have an appropriate level of expertise to confirm that it is of an acceptable scientific standard.

# Reviewer report: Reviewer response for version 1

**DOI:** 10.5256/f1000research.180079.r414422

**Published:** 2025-10-15

**Authors:** Colleen Aldous

**Affiliations:** 1 University of Kwa-Zulu Natal, Durban, South Africa

**1. Clarity of Presentation and Use of Current Literature — *Partly***

 The manuscript is clearly structured but requires significant improvement in linguistic precision, conceptual coherence, and literature contextualization. Numerous grammatical errors, awkward phrasing, and inconsistent terminology (e.g., “precised medicine,” “pawing inroads”) reduce readability and professionalism. The introduction conflates *precision*, *personalised*, and *genomic* medicine without defining them distinctly.

**Recommendations:**

- The paper should undergo **comprehensive language and style editing** by a professional editor fluent in academic English.
- Expand the **literature review** to include international studies on public awareness of precision medicine from Europe, North America, and East Asia to enable meaningful benchmarking.
- Provide clearer definitions of key terms and maintain terminological consistency throughout the text.
- Avoid promotional or uncritical language (e.g., “UAE, leading in adapting new technologies”), and instead adopt an evidence-based tone.

**Essential for scientific soundness:** Clarity, precision in terminology, and a more balanced, globally contextualised literature review.

**2. Study Design and Technical Soundness — *Partly***

 The cross-sectional, online, snowball-sampled design is acceptable for exploratory research but suffers from severe **sampling bias**. The sample (94% under 50, 60% with bachelor’s degrees, nearly half students) is not representative of the UAE general population. The methodology lacks depth in survey validation and data analysis transparency.

**Recommendations:**

- Explicitly acknowledge **selection bias and lack of representativeness** in the discussion and conclusions.
- Describe **survey recruitment** channels and mechanisms to prevent duplicate responses.
- Provide **validation metrics** for the questionnaire (e.g., Cronbach’s alpha, pilot testing results).
- Justify the scoring systems for “knowledge,” “acceptance,” and “practice” or reference validated instruments used in prior research.

**Essential for scientific soundness:** Clearer methodological transparency and acknowledgement of sampling limitations.

**3. Replicability of Methods and Analysis — *No / Partly***

 The methods section lacks the detail necessary for replication. The full questionnaire, item wording, coding procedures, and scoring logic are not included. The statistical methods are outlined but not sufficiently justified or described.

**Recommendations:**

- Include the **complete survey instrument** as a supplementary appendix.
- Provide a **data dictionary and scoring codebook** explaining how raw responses were converted into numerical scores.
- Specify **handling of missing data**, **normality testing**, and any data cleaning procedures.
- Upload **SPSS syntax or analytical scripts** to the data repository for transparency.

**Essential for scientific soundness:** Full methodological documentation and explicit variable coding details.

**4. Statistical Analysis and Interpretation — *Partly***

 The use of Mann–Whitney U and Kruskal–Wallis tests is appropriate for ordinal data, but the analytical plan is limited to univariate tests without adjustment for confounders or multiple comparisons. The interpretation overstates weak or marginal associations.

**Recommendations:**

- Include **multivariate regression models** to control for key demographic confounders (age, gender, education, income).
- Report **effect sizes and confidence intervals** to contextualise significance levels.
- Apply **correction for multiple comparisons** to reduce false-positive results.
- Refrain from labelling non-significant trends (e.g., *p* = 0.056) as meaningful.
- Ensure interpretation distinguishes between **statistical** and **clinical** significance.

**Essential for scientific soundness:** More robust inferential analysis and moderation of interpretive claims.

**5. Data Availability and Reproducibility — *Partly***

 Although the dataset is deposited in Harvard Dataverse, its utility is limited by insufficient documentation. The repository lacks variable definitions, questionnaire content, and analytical code.

**Recommendations:**

- Provide a **complete metadata package**, including variable labels, coding scheme, and questionnaire text.
- Include **derived variable computation formulas** for transparency (e.g., how familiarity or practice scores were calculated).
- Attach **SPSS syntax or scripts** used for analysis.
- Verify that all shared data are **fully anonymised** and ethically compliant.

**Essential for scientific soundness:** Complete, well-documented dataset and analytical transparency.

**6. Conclusions and Interpretation — *Partly***

 The conclusions are **overstated** relative to the data. The results indicate limited awareness and engagement, yet the discussion portrays a “moderately aware” and “positively inclined” population. This optimistic framing is not supported by the evidence.

**Recommendations:**

- Temper conclusions to reflect **exploratory scope** and **descriptive findings**.
- Explicitly acknowledge the **limitations of generalizability** due to sampling bias and self-selection.
- Avoid implying **causal relationships** (e.g., chronic disease status causing acceptance).
- Reframe recommendations as **hypothesis-generating** rather than prescriptive policy guidance.

**Essential for scientific soundness:** Conclusions must align proportionally with the strength of evidence.

**Overall Summary**

 This paper addresses a relevant topic in precision medicine awareness, but is **methodologically weak, under-analysed, and over-interpreted**. It would not meet the publication standards of a high-impact journal in its current form. To make it scientifically sound, the authors must:

1. Conduct professional language and structural editing.
2. Expand and deepen the literature review.
3. Fully document methods, questionnaire, and data processing.
4. Strengthen the statistical analysis with multivariate and effect-size reporting.
5. Revise the discussion and conclusions to accurately reflect descriptive findings.
6. Ensure full data reproducibility and transparency through detailed documentation.

Only after these revisions can the manuscript be considered methodologically credible and analytically defensible.

Is the work clearly and accurately presented and does it cite the current literature?

Partly

If applicable, is the statistical analysis and its interpretation appropriate?

Partly

Are all the source data underlying the results available to ensure full reproducibility?

Partly

Is the study design appropriate and is the work technically sound?

Partly

Are the conclusions drawn adequately supported by the results?

Partly

Are sufficient details of methods and analysis provided to allow replication by others?

No

Reviewer Expertise:

NA

I confirm that I have read this submission and believe that I have an appropriate level of expertise to confirm that it is of an acceptable scientific standard, however I have significant reservations, as outlined above.

# Response

**Published:** 2025-11-19

**Authors:** Jisha Myalil Lucca

**Affiliations:** Pharmacy Practice, Gulf medical University, Ajman, United Arab Emirates

**Response to Reviewer Comment:**

 We sincerely thank the reviewer for the constructive feedback regarding the clarity, linguistic precision, and conceptual framing of the manuscript. We have carefully revised the paper to address these concerns in the following ways:

**Language and Grammar:**

 All sections of the manuscript have been thoroughly edited to correct grammatical errors, remove awkward phrasing, and improve overall readability and flow. We have also ensured consistency in terminology throughout (e.g., replaced “precised medicine” with “precision medicine” and removed unclear phrases such as “pawing inroads”).

**Conceptual Coherence:**

  A definition of all this terminology is added in the manuscript **precision medicine**, **personalized medicine**, and Pharmacogenomics

**Literature Contextualization:**

 Additional and more recent references have been incorporated to better situate the study within current literature and to strengthen the rationale for exploring public awareness and acceptance of precision medicine in the UAE.

 Essential for scientific soundness: Clarity, precision in terminology, and a more balanced, globally contextualised literature review.

 We fully acknowledge the reviewer’s emphasis on enhancing the scientific soundness of the manuscript. Accordingly, we have implemented the following revisions:

**Clarity and Precision in Terminology:**

 Key terms such as *precision medicine*, *personalized medicine*, and *genomic medicine* have been clearly defined and consistently used throughout the manuscript to ensure conceptual precision and avoid ambiguity.

**Study Design and Technical Soundness — *Partly***

**The cross-sectional, online, snowball-sampled design is acceptable for exploratory research but suffers from severe sampling bias. The sample (94% under 50, 60% with bachelor’s degrees, nearly half students) is not representative of the UAE general population. The methodology lacks depth in survey validation and data analysis transparency.**

**Response:**

 We thank the reviewer for these insightful comments regarding the study design, sampling method, and data validation. We have carefully addressed each concern as outlined below:

1. **Acknowledgment of Sampling Bias and Representativeness:**
  We agree that the snowball-sampled, online survey may not fully represent the UAE’s general population  we have **added a new session limitation of study and  acknowledged this limitation**
2. **Survey Recruitment and Duplicate Response Control:**
  We have now provided a **clearer description of the recruitment process** in the *Methods* section, specifying the online platforms  and methods specifically used to minimize duplicate entries, the **Google Forms “one response per device” restriction** was enabled, and responses with identical IP addresses or missing consent were excluded during data cleaning.
3. **Validation Metrics and Instrument Reliability:**
  We thank the reviewer for this valuable observation. In the revised manuscript, we have now provided the full questionnaire as supplementary material. The internal consistency of the questionnaire was assessed to ensure reliability of the data collected. The section on participants’ knowledge of precision medicine demonstrated excellent reliability, with a Cronbach’s alpha of 0.852. Similarly, the section evaluating utilization patterns of precision medicine services showed a Cronbach’s alpha of 0.855, indicating high internal consistency. These values suggest that the questionnaire items were well-correlated and consistently measured the intended constructs, supporting the reliability of the study findings.   A similar statements is added in the methodology session Finally, we have expanded the discussion to acknowledge the potential sampling bias associated with the snowball sampling approach.
4. **Justification of Scoring Systems:**
  Scoring methods for *knowledge*, *acceptance*, and *practice* were developed in alignment with approaches used in prior research assessing awareness and perceptions of precision medicine (e.g., Edris et al., 2023; Almaazmi et al., 2022). We have added citations and a short explanatory paragraph in the *Methods* section clarifying how Likert-scale items were aggregated and interpreted to create composite scores.
5. **Replicability of Methods and Analysis — *No / Partly***
  The methods section lacks the detail necessary for replication. The full questionnaire, item wording, coding procedures, and scoring logic are not included. The statistical methods are outlined but not sufficiently justified or described.
**Recommendations:**

- Include the **complete survey instrument** as a supplementary appendix.
- Provide a **data dictionary and scoring codebook** explaining how raw responses were converted into numerical scores.
- Specify **handling of missing data**, **normality testing**, and any data cleaning procedures.
- Upload **SPSS syntax or analytical scripts** to the data repository for transparency.
- **Essential for scientific soundness:** Full methodological documentation and explicit variable coding details.
- The complete questionnaire, including all item wordings and response options, has been provided as a supplementary appendix to facilitate replication. A statement of scoring codebook and data cleaning is added in the methods The rationale for each statistical test has been clarified in the Methods section.

**Data Availability and Reproducibility — *Partly***

**Response: **We appreciate the reviewer’s suggestion to include multivariate regression analyses and adjustments for multiple comparisons. While we acknowledge that such analyses could provide more robust inferential insights, the current study is an **undergraduate-level exploratory project**, and the available sample size and scope limit our ability to perform these analyses comprehensively.

 We have, however, strengthened the rigor of our analysis by:

 Using **appropriate non-parametric tests** (Mann–Whitney U and Kruskal–Wallis) for ordinal and non-normally distributed data.

 Reporting **effect sizes where feasible** and clearly distinguishing between statistical significance and non-significant trends.

 Providing detailed descriptions of **scoring procedures, variable coding, and data cleaning** in the Methods section.

**Conclusions and Interpretation — *Partly***

**Response: **We have revised the conclusions to reflect the exploratory and descriptive nature of the study by tempering language to “limited to modest awareness,” acknowledging the non-representative sample, reframing recommendations as areas for future research, and avoiding causal claims, thereby ensuring alignment with the strength of evidence.

# Reviewer report: Reviewer response for version 1

**DOI:** 10.5256/f1000research.180079.r414418

**Published:** 2025-09-29

**Authors:** Chibuzor F. Ogamba

**Affiliations:** 1 University of Oxford, Oxford, UK

The manuscript would benefit from substantial methodological clarification and tighter statistical reporting to be suitable for indexing.

 The authors report on public awareness, acceptance and accessibility of precision medicine in the UAE using a cross-sectional study design. About half of the sample reported at least some familiarity with precision medicine terminology. Group differences were tested using non-parametric methods.

 Strengths: Focused, policy-relevant question

 Methods reporting is incomplete and hampers replicability: 

 - Define all constructs (e.g., familiarity, knowledge, acceptance, perceived benefits, preferences for pharmacogenetic testing) and how each score is computed.

 - Move any construct definitions currently in Results into Methods.

 - Clarify whether the sample is intended to be population-representative; if not, temper generalizations.

 - The statement “Gender differences approached statistical significance (p = 0.056) … suggesting a potential trend” overstates evidence and is misleading at α=0.05. Moreover, the reported medians are identical (female 8; male 8), contradicting “slightly higher median.”

 - ‘familiarity’ vs ‘knowledge’ appear to be used interchangeably. Please state whether they are distinct constructs (different items/scales) or synonyms. If distinct, report separate definitions, scoring, and reliability.

 - Phrases such as “median around 5” are not informative without the scale range and anchors. Always report the possible range so readers can interpret the neutrality or magnitude accurately.

 - If baseline awareness/engagement with precision medicine is limited, respondents may not fully understand insurance-coverage items. Discuss construct validity and potential measurement error in the Limitations.

 - Regarding the questionnaire, the authors do not cite what sources the existing questionnaire was adapted from. 

 -“Panel of five experts” includes two pharmacy students; that’s not an expert panel.

 - “Given for a common man” is informal, non-inclusive wording, and describes neither the methods nor the sample  nor what feedback was gathered

 - Limitations section is currently missing.

 - In Figure 3, clarify in the Methods and the figure caption whether multiple selections were allowed. If yes, state “multiple responses permitted; percentages may sum to >100%,” and avoid phrasing like “For 18% of respondents, the key purpose was cheaper treatment” which implies mutual exclusivity.

 - Consider aligning reporting with STROBE (checklist for cross-sectional studies):

 - Report exact p values to three decimals where possible (e.g., p = 0.056); use “p < 0.001” for very small values. Replace any “p < 0.5” with the exact value.

 - The Methods say “means ± SD for continuous variables,” but no means are presented. Either (a) present means ± SD (with normality justification), or (b) revise Methods to reflect medians (IQR) for skewed/ordinal data.

 - State the distribution of the outcomes eg outcomes are ordinal or non-normal that justifies choice of non-parametric tests.

 - Ensure percentages and frequencies follow a single style (e.g., “n (%)”) throughout.

Is the work clearly and accurately presented and does it cite the current literature?

Partly

If applicable, is the statistical analysis and its interpretation appropriate?

No

Are all the source data underlying the results available to ensure full reproducibility?

No

Is the study design appropriate and is the work technically sound?

No

Are the conclusions drawn adequately supported by the results?

No

Are sufficient details of methods and analysis provided to allow replication by others?

No

Reviewer Expertise:

Epidemiology

I confirm that I have read this submission and believe that I have an appropriate level of expertise to state that I do not consider it to be of an acceptable scientific standard, for reasons outlined above.

# Response

**Published:** 2025-11-19

**Authors:** Jisha Myalil Lucca

**Affiliations:** Pharmacy Practice, Gulf medical University, Ajman, United Arab Emirates

We sincerely thank the reviewer for the constructive feedback

 Methods reporting is incomplete and hampers replicability: - Define all constructs (e.g., familiarity, knowledge, acceptance, perceived benefits, preferences for pharmacogenetic testing) and how each score is computed. 

***Response: **We have now clearly defined all key constructs in the Methods section.*

 Move any construct definitions currently in Results into Methods.

***Response: *** Done

 The statement “Gender differences approached statistical significance (p = 0.056) … suggesting a potential trend” overstates evidence and is misleading at α=0.05. Moreover, the reported medians are identical (female 8; male 8), contradicting “slightly higher median.”

***Response: **We acknowledge the reviewer’s comment and have revised the manuscript accordingly.*

 ‘familiarity’ vs ‘knowledge’ appear to be used interchangeably. Please state whether they are distinct constructs (different items/scales) or synonyms. If distinct, report separate definitions, scoring, and reliability.

***Response: **In our study, “familiarity” and “knowledge” are used as synonyms*

 Phrases such as “median around 5” are not informative without the scale range and anchors. Always report the possible range so readers can interpret the neutrality or magnitude accurately.

***Response: **The single instance in which the median was previously described as “around 5” has now been revised to include the full scale range and context. All other medians in the manuscript are already reported with interquartile ranges, ensuring clarity and interpretability, and therefore no additional modifications are required.*

 - If baseline awareness/engagement with precision medicine is limited, respondents may not fully understand insurance-coverage items. Discuss construct validity and potential measurement error in the Limitations. 

***Response: **Updated the limitation* 

 Regarding the questionnaire, the authors do not cite what sources the existing questionnaire was adapted from. 

***Response: **The questionnaire was adapted from previously published studies on public awareness and perceptions of precision medicine which has now been explicitly cited in the Methods section.*

 -“Panel of five experts” includes two pharmacy students; that’s not an expert panel.

***Response: **Regarding the expert panel, we acknowledge that including pharmacy students does not fully meet the traditional definition of “experts”; however, as they are master’s-level students, they were considered semi-experts, and this clarification has been updated in the Methods section.*

 - “Given for a common man” is informal, non-inclusive wording, and describes neither the methods nor the sample  nor what feedback was gathered

***Response: **The questionnaire was tested with individuals from the general public, specifically one investigator family member  to assess clarity, comprehensibility, and ease of completion. Participants were asked whether any items were difficult to understand or ambiguous.*

 Limitations section is currently missing:

***Response: **Added*

  In Figure 3, clarify in the Methods and the figure caption whether multiple selections were allowed. If yes, state “multiple responses permitted; percentages may sum to >100%,” and avoid phrasing like “For 18% of respondents, the key purpose was cheaper treatment” which implies mutual exclusivity.

***Response: **Figure 3 caption have been updated to clarify that multiple responses were permitted, and percentages may sum to more than 100%.*

 Consider aligning reporting with STROBE (checklist for cross-sectional studies):

 The manuscript has been reviewed and revised to align with the STROBE checklist for cross-sectional studies.

***Response:*** *Relevant sections, including study design, participant recruitment, variables, data sources, statistical methods, and limitations, have been updated to improve clarity, transparency, and completeness of reporting.*

 Report exact p values to three decimals where possible (e.g., p = 0.056); use “p < 0.001” for very small values. Replace any “p < 0.5” with the exact value. *Done*

 The Methods say “means ± SD for continuous variables,” but no means are presented. Either (a) present means ± SD (with normality justification), or (b) revise Methods to reflect medians (IQR) for skewed/ordinal data.

***Response:*** *Modification made in methodology*

 Ensure percentages and frequencies follow a single style (e.g., “n (%)”) throughout.

***Response: **Modification done in results*

# Reviewer report: Reviewer response for version 1

**DOI:** 10.5256/f1000research.180079.r397406

**Published:** 2025-08-27

**Authors:** Mohamed Zahir Alimohamed

**Affiliations:** 1 Muhimbili University of Health and Allied Sciences, Dar es Salaam, Tanzania

This cross-sectional study evaluates awareness, acceptance, and utilization of precision medicine among the general population in the UAE using an online questionnaire. The authors present descriptive and inferential statistics to examine associations between sociodemographic variables and awareness or practice of precision medicine. The article highlights low engagement with genetic testing, limited awareness of insurance coverage, and moderate familiarity with precision medicine terms. The study concludes with recommendations for improving public engagement.

 Areas for Improvement

1. Clarity and Writing Quality (Partly): The manuscript suffers from language and grammar issues, especially in the introduction and methods sections. Recommendation: A thorough language edit by a native English speaker or professional editor is required.
2. Incomplete Literature Context (Partly): While the study cites relevant and recent literature, it lacks a deeper discussion of similar public awareness studies from other regions or global comparisons. Recommendation: Strengthen the introduction and discussion by incorporating more international studies on public awareness of precision medicine for better benchmarking.
3. Methodological Transparency (Partly): The sampling approach (snowball sampling) is appropriate for exploratory work but introduces bias. This limitation is not discussed sufficiently. The survey’s validation process is briefly mentioned, but the full questionnaire is not provided in the supplementary data. It is unclear how the knowledge, acceptance, and practice scores were calculated and whether internal consistency (e.g., Cronbach’s alpha) was assessed. Recommendation: Clearly outline the scoring methodology, include the full questionnaire as supplementary material, and address sampling limitations in the discussion.
4. Conclusions Not Fully Supported (Partly): While the data support the claim of limited awareness and utilization, the conclusion suggests "generally positive perception" which is not robustly demonstrated in the results. Recommendation: Temper conclusions to align with the modest awareness and engagement levels shown by the data.

Final Recommendation

 The study addresses a meaningful topic and presents original data from a region underrepresented in public genomics research. However, substantial revisions are necessary, primarily in clarity, methodology transparency, and conclusion framing before it is suitable for indexing.

Is the work clearly and accurately presented and does it cite the current literature?

Partly

If applicable, is the statistical analysis and its interpretation appropriate?

Yes

Are all the source data underlying the results available to ensure full reproducibility?

Yes

Is the study design appropriate and is the work technically sound?

Yes

Are the conclusions drawn adequately supported by the results?

Partly

Are sufficient details of methods and analysis provided to allow replication by others?

Partly

Reviewer Expertise:

Molecular Genetics, Genetic Counselling, Molecular diagnostics, Cardiomyopathies, SCD, NCDs, Precision Medicine.

I confirm that I have read this submission and believe that I have an appropriate level of expertise to confirm that it is of an acceptable scientific standard, however I have significant reservations, as outlined above.

# Response

**Published:** 2025-11-19

**Authors:** Jisha Myalil Lucca

**Affiliations:** Pharmacy Practice, Gulf medical University, Ajman, United Arab Emirates

Thank you for your valuable review and constructive feedback, which will help us strengthen our study and its recommendations.

 Areas for Improvements

- Clarity and Writing Quality (Partly): The manuscript suffers from language and grammar issues, especially in the introduction and methods sections. Recommendation: A thorough language edit by a native English speaker or professional editor is required.
- ***Response: *** We *appreciate your feedback, and the manuscript has been thoroughly revised for language and grammar using various online editing tools to improve clarity and quality.*
- Incomplete Literature Context (Partly): While the study cites relevant and recent literature, it lacks a deeper discussion of similar public awareness studies from other regions or global comparisons. Recommendation: Strengthen the introduction and discussion by incorporating more international studies on public awareness of precision medicine for better benchmarking.
- ***Response: **Thank you for the suggestion; we have strengthened the introduction and discussion by incorporating additional international studies on public awareness of precision medicine to provide better global context and benchmarking.*
- Methodological Transparency (Partly): The sampling approach (snowball sampling) is appropriate for exploratory work but introduces bias. This limitation is not discussed sufficiently. The survey’s validation process is briefly mentioned, but the full questionnaire is not provided in the supplementary data. It is unclear how the knowledge, acceptance, and practice scores were calculated and whether internal consistency (e.g., Cronbach’s alpha) was assessed. Recommendation: Clearly outline the scoring methodology, include the full questionnaire as supplementary material, and address sampling limitations in the discussion.
- ***Response: **We thank the reviewer for this valuable observation. In the revised manuscript, we have now provided the full questionnaire as supplementary material. The internal consistency of the questionnaire was assessed to ensure reliability of the data collected. The section on participants’ knowledge of precision medicine demonstrated excellent reliability, with a Cronbach’s alpha of 0.852. Similarly, the section evaluating utilization patterns of precision medicine services showed a Cronbach’s alpha of 0.855, indicating high internal consistency. These values suggest that the questionnaire items were well-correlated and consistently measured the intended constructs, supporting the reliability of the study findings.   A similar statement is added in the methodology session Finally, we have expanded the discussion to acknowledge the potential sampling bias associated with the snowball sampling approach.*
- Conclusions Not Fully Supported (Partly): While the data support the claim of limited awareness and utilization, the conclusion suggests "generally positive perception" which is not robustly demonstrated in the results. Recommendation: Temper conclusions to align with the modest awareness and engagement levels shown by the data.
- ***Response: **Thank you for this valuable comment. We agree that the conclusion required refinement to more accurately reflect the study findings. Accordingly, we have revised the conclusion*
